# Health Care Providers’ Awareness, Knowledge, and Readiness to Implement Digital Cognitive Assessments in Primary Care Settings

**DOI:** 10.1093/geroni/igaf122.4252

**Published:** 2025-12-31

**Authors:** Jennifer Pettis, Lisa McGuire, Ben Tiede, Emily Scholler

**Affiliations:** Gerontological Society of America, Washington, District of Columbia, United States; Gerontological Society of America, Atlanta, Georgia, United States; Global CEO Initiative on Alzheimer’s Disease, Wayne, Pennsylvania, United States; Global CEO Initiative on Alzheimer’s Disease, Wayne, Pennsylvania, United States

## Abstract

The growing number of people with undiagnosed dementia underscores the urgent need to improve routine screening procedures for cognitive impairment in older adults. While health care continues to advance technologically, most traditional cognitive screening tools remain paper based. Emerging research suggests that the use of digital cognitive assessments (DCAs) could improve the detection of cognitive impairment while easing the burden on primary care teams who are already stretched thin. To effectively prepare primary care teams to implement DCAs, it is important to understand their current awareness, knowledge, and readiness to integrate DCAs into their clinical workflows. The Global CEO Initiative on Alzheimer’s Disease (CEOi) is leading a global effort to advance the adoption of DCAs in clinical care, aiming to provide patients with a simpler, timelier, and more accurate diagnostic experience. As part of this effort, CEOi commissioned the Gerontological Society of America (GSA) and the American Academy of Family Physicians (AAFP) to survey their members in August and September 2025. The survey explored health care providers’ awareness, knowledge, and readiness to use DCAs in primary care settings. Findings from this survey offer key insights to guide recommendations on the information, training, and resources needed to equip the primary care workforce for early detection of cognitive impairment through the utilization of DCAs. Attendees of this session will gain an understanding of the survey results and learn what supports health care providers identified as necessary to effectively integrate DCAs into their primary care practices.

